# Mobile Health Application for the Prevention of Stroke (MAPS): a pilot, single-arm study on an innovative health application designed for primary stroke prevention

**DOI:** 10.1186/s12883-025-04560-3

**Published:** 2026-01-10

**Authors:** Matthias N. Ungerer, Kilian Reichel, Manuel Feisst, Christine Tunkl, Benjamin Quenzer, Christoph Gumbinger

**Affiliations:** 1 Department of Neurology, University Hospital Heidelberg, Heidelberg, Germany; 2 Institute of Medical Biometry, Heidelberg University, Heidelberg, Germany; 3 Netzgut GmbH, Heidelberg, Germany

**Keywords:** Primary prevention, Habits, Mobile applications

## Abstract

**Background:**

Long-term changes in healthy behavior are more likely when small improvements are made in daily health habits. Health applications can serve as cost-effective tools to help people adopt healthier lifestyles. In response to this need, we developed the MAPS app - an easy-to-use and innovative mobile health app for the primary prevention of stroke.

**Methods:**

We conducted a prospective, pre-post, single-arm, multicomponent interventional pilot study over 6 months to evaluate the ability of the MAPS app to enhance healthy habits and reduce stroke risk factors among a healthy adult population recruited from an urban employer in Germany. Daily health habits were assessed using questionnaires at the beginning of the study, at 3 months, and at 6 months. We also measured estimated stroke risk over 5 and 10 years, as well as systolic blood pressure, Body Mass Index (BMI), abdominal circumference, LDL cholesterol, and HbA1c levels at the beginning and after 6 months.

**Results:**

A total of 35 participants were recruited. One participant was lost to follow-up. Our analysis showed significant improvements in the daily health habits questionnaire after 6 months (mean change of 5.6 points; 95% confidence interval (CI) 3.20 to 7.95). We observed significant reductions in the estimated 5-year stroke risk (mean change − 0.3%; 95% CI -0.52 to -0.04), BMI (mean change − 0.6 kg/m²; 95% CI -1.17 to -0.02), and abdominal circumference (mean change − 2.8 cm; 95% CI -5.28 to -0.25). Changes in LDL cholesterol and HbA1c levels were not significant. Male participants tended to have lower initial scores on daily habit questionnaires, particularly in dietary health choices, and benefited more from participation in the study.

**Conclusions:**

Our results suggest that the MAPS app may help promote healthier daily habits and reduce stroke risk. While promising, these preliminary findings require further validation through larger randomized controlled trials. Additionally, they indicate potential gender-specific responses that need more investigation.

**Trial registration:**

The study was registered retrospectively in the German Clinical Trials Register on 25th November 2024. (DRKSID DRKS00034491). Trial registration was submitted before enrollment started, but it was finalized after recruitment began due to technical issues.

**Supplementary Information:**

The online version contains supplementary material available at 10.1186/s12883-025-04560-3.

## Introduction

Stroke is one of the leading causes of death and disability worldwide [1]. In addition to optimizing the treatment of acute strokes through intravenous thrombolysis and mechanical thrombectomy, there is an increasing focus on improving primary and secondary stroke prevention [2]. By reducing and treating arterial hypertension, hyperlipoproteinemia, diabetes mellitus, and obesity, as well as promoting a healthy lifestyle and regular physical activity, the incidence of stroke and the risk of recurrent stroke can be significantly reduced [3]. Research indicates that addressing multiple concurrent modifiable risk factors at once may be more effective in reducing overall mortality risk. However, most interventions have typically focused on individual risk factors [4–6]. Primary prevention campaigns utilizing educational initiatives can successfully raise awareness of modifiable risk factors and therefore promote a healthier lifestyle [7]. New insights from behavioral research suggest that long-term behavioral changes are better achieved through small changes in participants’ daily routines, particularly in the case of physical activity [8]. This concept is also increasingly used in fitness apps. The development of smartphone-based apps to improve so-called “daily habits” represents a cost-effective technology with great potential in the healthcare sector and can be used for primary stroke prevention.

However, the effects of app-based health interventions have thus far been modest, and more trials are needed to improve the evidence for these interventions [9]. Demonstrating efficacy in primary prevention studies may be further complicated by the recruitment of younger and more health-conscious participants. A meta-analysis of four large randomized controlled trials conducted on healthy young adults did not find conclusive evidence favoring app-based interventions [10]. Overall, there is an evidence gap and mixed findings regarding the effectiveness of app-based interventions for primary prevention in young healthy adults. We therefore aimed to generate additional evidence supporting the use of app-based interventions as a primary stroke prevention strategy. There is also a lack of consensus on how to measure the success of app-based interventions [11]. This is attributable to interventions addressing different unhealthy habits, multiple potential outcome measures, uncertainties regarding the appropriate follow-up time, differences in target populations, and challenges in implementation (e.g., adherence to interventions and healthcare system integration). We conducted this single-arm pilot study as a first step to assess our study design, evaluate the intervention’s potential effect, and generate hypotheses for future larger randomized trials.

We aimed to leverage insights from behavioral research to promote a healthy lifestyle and reduce cerebrovascular risk factors. To this end, we developed a lifestyle app together with an industry partner, which was evaluated in a study involving healthy working-age participants. The goal of this exploratory pilot study was to evaluate feasibility and engagement, and to estimate preliminary effects of this lifestyle app on daily health habits and stroke risk markers. We hypothesized improvements in daily habits assessed by questionnaire (primary hypothesis) as well as improvements in stroke risk scores and other objective risk factors (secondary hypothesis) at 6 months.

## Materials and methods

### Study design, setting, and population

We conducted a single-arm, prospective, pre-post app-based intervention study in a healthy population over a period of 6 months between July 1, 2024, and December 31, 2024. The intervention utilized a multicomponent design that included training for participants, feedback letters following physical examinations, and the use of the MAPS app. The intervention period was set at six months, as habit formation can typically take anywhere from several weeks up to six months [12]. Additionally, previous studies have shown that cerebrovascular risk factors, such as blood pressure and body weight, can be effectively modified during six-month interventions [13, 14]. Participants were primarily recruited from a local urban municipal employer with a predominantly sedentary workforce in Germany. The local municipal employer was unrelated to the company that developed the MAPS app. As this was an exploratory pilot study, no formal sample size calculation was performed, but the sample size was chosen for feasibility reasons. We aimed for a sample size of 30 participants, which is standard in pilot trials with continuous outcomes [15]. Participants were selected using a convenience sampling method.

### Eligibility criteria

All participants needed to be between 18 and 70 years old, own an Android smartphone with a pedometer function, and provide written consent. They were required to have the physical and psychological ability to engage in moderate physical activity, as confirmed by a physical examination. We excluded individuals with at least moderate disability who required some help to perform daily activities (modified Rankin Scale >2) or those who intended to become pregnant during the study period [16]. 

### Ethics approval

Informed written consent for participation was obtained from all participants. Ethical clearance was obtained from the ethics committee of Heidelberg University (S-685/2023). All participants were provided free access to our MAPS app and received a small compensation for expenses for attending the second physical examination at six months. Participation was voluntary and was encouraged by the employer. Netzgut GmbH was commissioned to develop a mobile app for data collection and processing for this project. A data processing agreement was established, incorporating measures in accordance with the Datenschutz-Grundverordnung, which is the German implementation of the General Data Protection Regulation. Netzgut GmbH subcontracted Hetzner Online AG to provide a bare-metal server. Access to the data was restricted to Netzgut GmbH employees, and the data was stored in a pseudonymized and encrypted format. Upon completion of the project, the data was sent to the project leaders through encrypted channels and was securely deleted from Netzgut GmbH’s servers.

### Data collection instruments

#### Daily habits

The questionnaires to assess daily habits consisted of three parts: A translated version of the Healthy Lifestyle and Personal Control Questionnaire (HLPCQ), questions necessary to estimate stroke risk using the Stroke Riskometer App™ (SRA™), and a limited number of additional questions (e.g., on the number of vegetable portions consumed per day). Most items were based on the HLPCQ questionnaire developed by Darviri et al., as published in 2014 [17]. This questionnaire was developed to evaluate lifestyle interventions and has been used and validated in various studies since its publication [18–20]. We did not make any additional alterations to the original questionnaire, which includes 26 questions assigned to 5 subcategories (Dietary Health Choices; Dietary Harm Avoidance; Daily Routine; Organized Physical Exercise; Social and Mental Balance), and assessed using a four-point Likert scale. Higher scores (range 26–104 points for the total score) indicate healthier daily habits [17]. The original questionnaire was translated by two native German speakers into a German version of the HLPCQ (gvHLPCQ). Changes in the gvHLPCQ were the primary outcome of our study. The SRA™ is a validated tool to assess the 5- and 10-year stroke risk promoted by the World Stroke Organization [21]. It demonstrated valid results in cross-cultural settings. It uses information on patient/medical history, lifestyle habits, and risk factors such as systolic blood pressure and Body Mass Index (BMI) to determine the individual stroke risk [22]. Additional features of the SRA™, including recommendations on stroke risk reduction, were not used in this study. SRA™ estimated stroke risk was determined for each participant at 0 and 6 months by combining survey data with the results from physical examinations at the beginning and end of this study.

#### Physical examinations

Clinical examinations involved assessing body weight, measuring abdominal circumference, and taking two blood pressure measurements in a standardized sequence. Blood pressure measurements were taken at the beginning and at the end of each physical examination, the average of which was used for statistical analysis. Body weight and blood pressure measurements were performed using the same digital automated devices for all participants. A venous blood sample was also collected during each examination to determine levels of low-density lipoprotein (LDL) cholesterol and hemoglobin A1c (HbA1c). All blood samples were taken after the participants had fasted.

### Data collection

Daily habits were assessed using a questionnaire at baseline (0), 3, and 6 months. The questionnaire administered at baseline can be found in the supplementary materials (Table S1). The three-month intermediate assessment aimed to identify early effects on daily habits and evaluate the sustainability of this change during the second half of the trial. Clinical examinations were conducted at the beginning and end of the study. Information on the usability of the app was collected in the questionnaire at 6 months. We also gathered data on user profiles to identify the most popular features of the app and to estimate user adherence. Participants were notified of their estimated stroke risks and other results from their physical examination by mail after each physical examination. Long-term follow-up data after the intervention were not collected.

### Development and design of the MAPS app

The app was developed by Netzgut GmbH based on specifications from the study team. The MAPS app was developed as a lifestyle application to encourage healthy habits. It did not need certification as a medical device according to national medical device regulations, as confirmed by the local ethics committee. The app features an appealing design and high user-friendliness to encourage regular use. It was designed exclusively for Android smartphones, with Netzgut GmbH as the app operator.

#### Functions and features

**Fig. 1:**
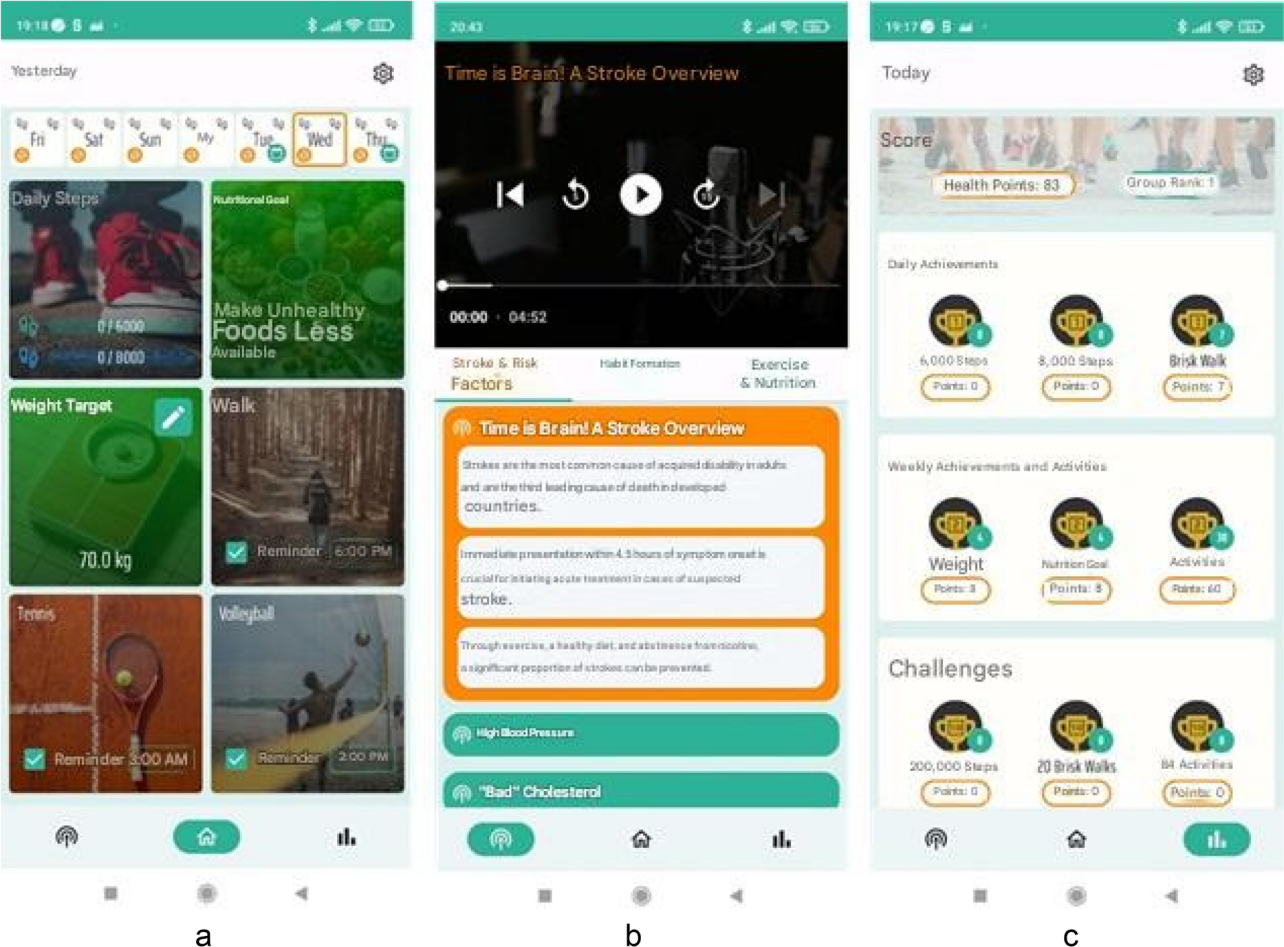
Illustration of the MAPS app translated from German to English, featuring three key components: **a** the main application interface, **b** the podcast interface, and **c** the health scorecard interface

During registration, users received privacy information about data collection and consent for accessing the smartphone’s pedometer function. After registration, users could access the app, which displayed daily and weekly goals along with the current step count (Fig. 1a). Users could set up to three additional daily activities from predefined options, e.g. cycling, swimming, walking, smoking cessation, or a self-defined activity. Users could select the frequency and schedule of activity reminders. Activities would automatically continue into the following week unless they were canceled. Users had to manually confirm activity completion. All participants were provided with simple and clear predefined weekly goals for healthy eating. For example, participants were advised to monitor the salt content of foods, cook with olive oil, eat whole-grain bread, eat fish twice a week, limit alcohol to one glass per day, or modify their kitchen environment to make unhealthy foods less accessible. Achievement of nutrition goals was to be confirmed daily. We also included a weekly manual entry of current body weight for the calculation of BMI. In the “Podcasts” window (Fig. 1b), users could access more than 20 podcasts on topics like “Stroke and Risk Factors,” “Habit Formation,” and “Sports and Nutrition.” The “Health Scorecard” (Fig. 1c) provided an overview of achievements with graphs and benchmarks against average cohort performance.

#### Feedback and reminder functions

Participants received feedback and reminders through push notifications. Positive feedback was provided for achieving individual daily and predefined weekly goals, completing challenges, and meeting step counts of 6,000 and 8,000. Daily reminders were sent at 8 AM on activities planned for a given day. Participants could set and schedule reminders for additional activities individually. Push notifications could be disabled in the “Settings” section.

#### Point system

A points system was designed to reward participants for their accomplishments, encouraging them to engage more actively. Participants earned two points for each completed planned activity, weekly weight entries, and daily nutrition goals met. They received one point for reaching daily step goals of 6,000 and 8,000 or for completing a power walk (1,000 steps in 10 min). The current point total was shown on the Health Scorecard. Completing specific challenges earned a one-time bonus of 20 points, and challenges could be completed repeatedly.

### Intervention

Before the study began, participants received online training on how to use the MAPS app and on the current guidelines-based recommendations on healthy eating, physical exercise, and the basic principles of habit formation. The online training session was made available online throughout the study period. All participants started the intervention on the same day and were requested to use the app over a period of 6 months. All participants had the freedom to use the app and its features as needed. There were no minimum usage requirements during the intervention, although we anticipated that the app would be used several times per week.

### Statistical analysis

Data were described using standard descriptive statistics. Results of individual survey items were described using the median and interquartile range (IQR) in cases when a Likert scale was used. Changes in individual survey items were analyzed using the Wilcoxon signed-rank test for paired data, while the Mann-Whitney U-test was applied to identify significant differences in the dependent variable between independent groups. Continuous variables were described using the mean ± standard deviation (SD). Percentages were rounded to one decimal point. P-values and Cohen’s d were rounded to three decimal points. Changes in gvHLPCQ scores, SRA™ scores, and the other objectifiable risk factors were examined using paired t-test analysis and described using mean differences and 95% confidence intervals (CI). The effect size was described using Cohen’s d. The difference between the change in gvHLPCQ scores between male and female participants was calculated using the independent t-test. Cronbach’s alpha was used to assess the internal consistency of the gvHLPCQ. Associations with the change in gvHLPCQ scores (0 to 6 months) were analyzed using univariate linear regression analysis and described using the Regression Coefficient B (RCB) and CI. Two-sided p-values < 0.05 were considered to indicate significant differences. We did not adjust for multiple testing since this was an exploratory study. All data collected were included in the statistical analysis of this study. In the case of missing data, analyses were performed only on participants for whom data were available (pairwise deletion). All analysis and figures were generated with IBM SPSS Statistics Version 29.0.2.0.

## Results

### Participant characteristics

**Fig. 2:**
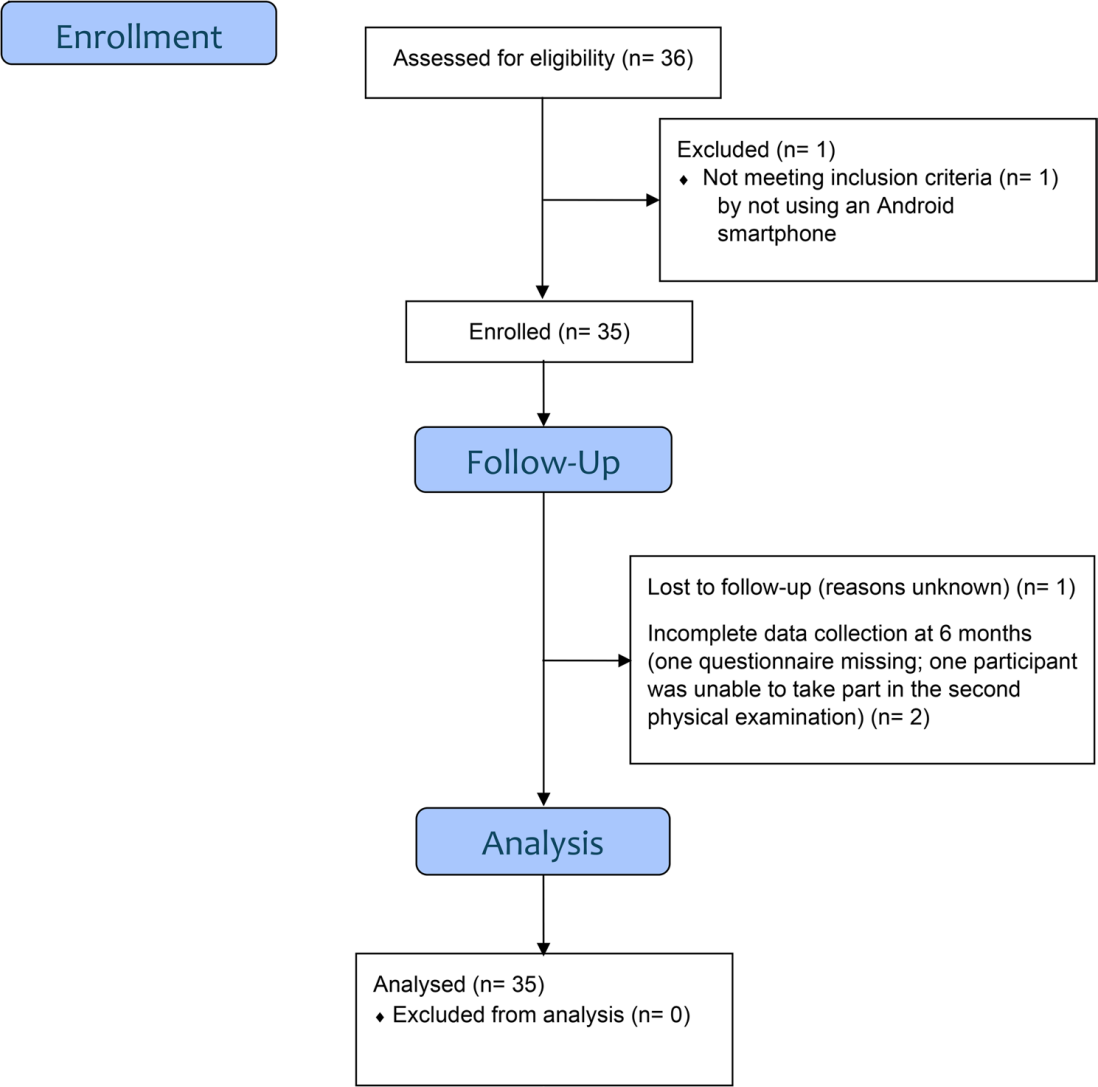
Consort flow diagram

A total of 35 participants were recruited for this study, meeting our predetermined goal of a minimum of 30 participants. There were no adverse events. One participant was lost to follow-up before the questionnaire at 3 months. Two participants provided incomplete data at the final examination at 6 months: one did not provide all the data of the questionnaire and the other did not take part in the second physical examination (see Fig. 2). There were no missing data for all other participants. Information regarding the demographics and risk factors of the participants is provided in Table 1.

**Table 1:** Demographics table on characteristics, cerebrovascular risk factors and medications used

Characteristics	Category	Frequency	Percentage
Gender	Male	7	20.0
	Female	28	80.0
	Total	35	100.0
Education	School graduation	1	2.9
	Professional apprenticeship	20	57.1
	University degree	14	40.0
	Total	35	100.0
Employment	Part-time	11	31.4
	Full-time	22	62.9
	Retired	2	5.7
	Total	35	100.0
Medication used	Category	Frequency	Percentage
Antihypertensive medication	Yes	7	20.0
	No	28	80.0
	Total	35	100.0
Cholesterol-lowering medication	Yes	2	5.7
	No	33	94.3
	Total	35	100.0
Risk factors	Category	Frequency	Percentage
Parent with history of stroke or myocardial infarction before age 65	Yes	10	28.6
	No	25	71.4
	Total	35	100.0
History of diabetes	Yes	3	8.6
	No	32	91.4
	Total	35	100.0
History of heart disease	Yes	4	11.4
	No	31	88.6
	Total	35	100.0
History of cardiomyopathy	Yes	0	0.0
	No	35	100.0
	Total	35	100.0
History of atrial fibrillation	Yes	1	2.9
	No	34	97.1
	Total	35	100.0
History of stroke or transient ischemic attack	Yes	0	0.0
	No	35	100.0
	Total	35	100.0

The mean age at the time of recruitment was 54 ± 7.7 years. Seven participants (20.0%) were male. Twenty-two worked full-time (62.9%) while 11 (31.4%) worked part-time. Two participants (5.7%) were retired. Twenty (57.1%) had completed an apprenticeship after schooling, 1 had a high school diploma and 14 (40.0%) participants had a college degree as their highest education. Mean LDL cholesterol values (120.9 ± 32.5 mg/dl), HbA1c (5.4 ± 0.6%), BMI (26.2 ± 4.9 kg/m²), abdominal circumference (96.2 ± 14.0 cm) and systolic blood pressure values (131.1 ± 14.0 mmHg) were within normal or slightly elevated ranges at the beginning of the study period. Three participants smoked tobacco daily (1–5 cigarettes in 2 cases, 16–20 in one case). Participants estimated that they had similar health habits when compared to their peers (median 2 [IQR 2–3], “similar”). Ten participants had a parent who experienced a stroke or myocardial infarction before the age of 65. Only two participants were on statins and 7 took antihypertensive medication at the beginning of the study. None of the participants had suffered a stroke or TIA. Twenty participants indicated that they had or were currently using fitness apps on their mobile devices.

When asked about the reasons that healthy habits were prohibited in the past, most participants indicated not having enough time (median 3 [IQR 2–4]; “often”) and predominating negative habits (median 3 [IQR 2–3]; “often”) as the leading issues followed by insufficient motivation (median 2 [IQR 2–3]; “sometimes”) and procrastination (median 2.5 [IQR 2–3.25]; ”sometimes”). Lack of awareness or knowledge of healthy habits was the least common factor (median 1 [IQR 1–2]; “rarely”).

### Main endpoints

#### Impact on gvHLPCQ Scores and estimated 5- and 10-years stroke risk

**Fig. 3:**
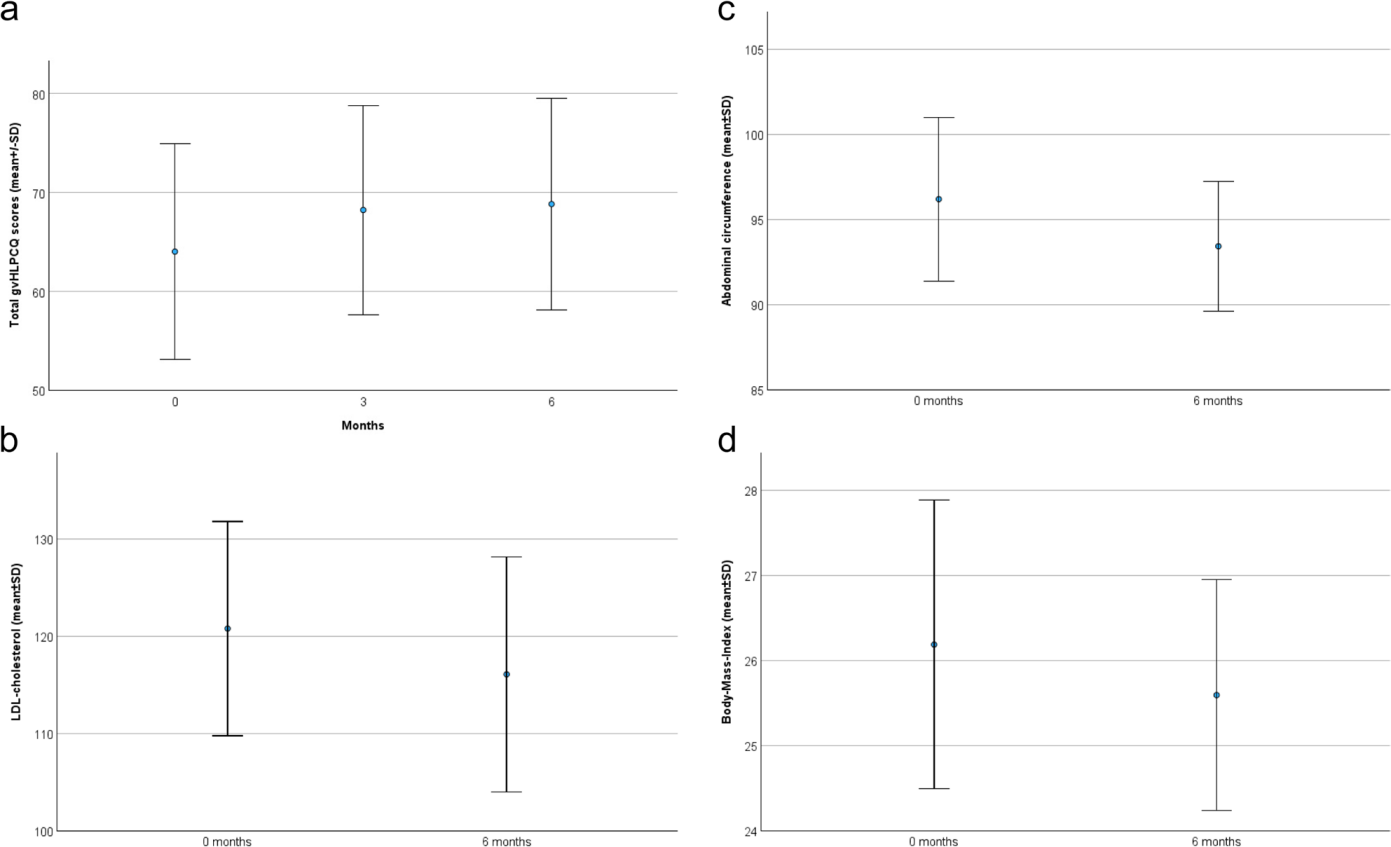
**A**: Total gvHLPCQ Scores at 0, 3 and 6 months. Abbreviations: gvHLPCQ German version of the Healthy Lifestyle and Personal Control Questionnaire, SD standard deviation, * missing data: 35 surveys at 0 months, 34 surveys at 3 months and 33 surveys at 6 months. **B**: LDL cholesterol (mg/dl) at 0 and 6 months. Abbreviations: LDL low-density lipoprotein, SD standard deviation, *missing data: 35 data points at 0 months, 34 data points at 6months. **C**: Abdominal circumference (cm) at 0 and 6 months. Abbreviations: SD standard deviation,*missing data: 35 data points at 0 months, 34 data points at 6months. **D**: Body-Mass-Index (kg/m²) at 0 and 6 months. Abbreviations: SD standard deviation, *missing data: 35 data points at 0 months, 34 data points at 6months

**Fig. 4:**
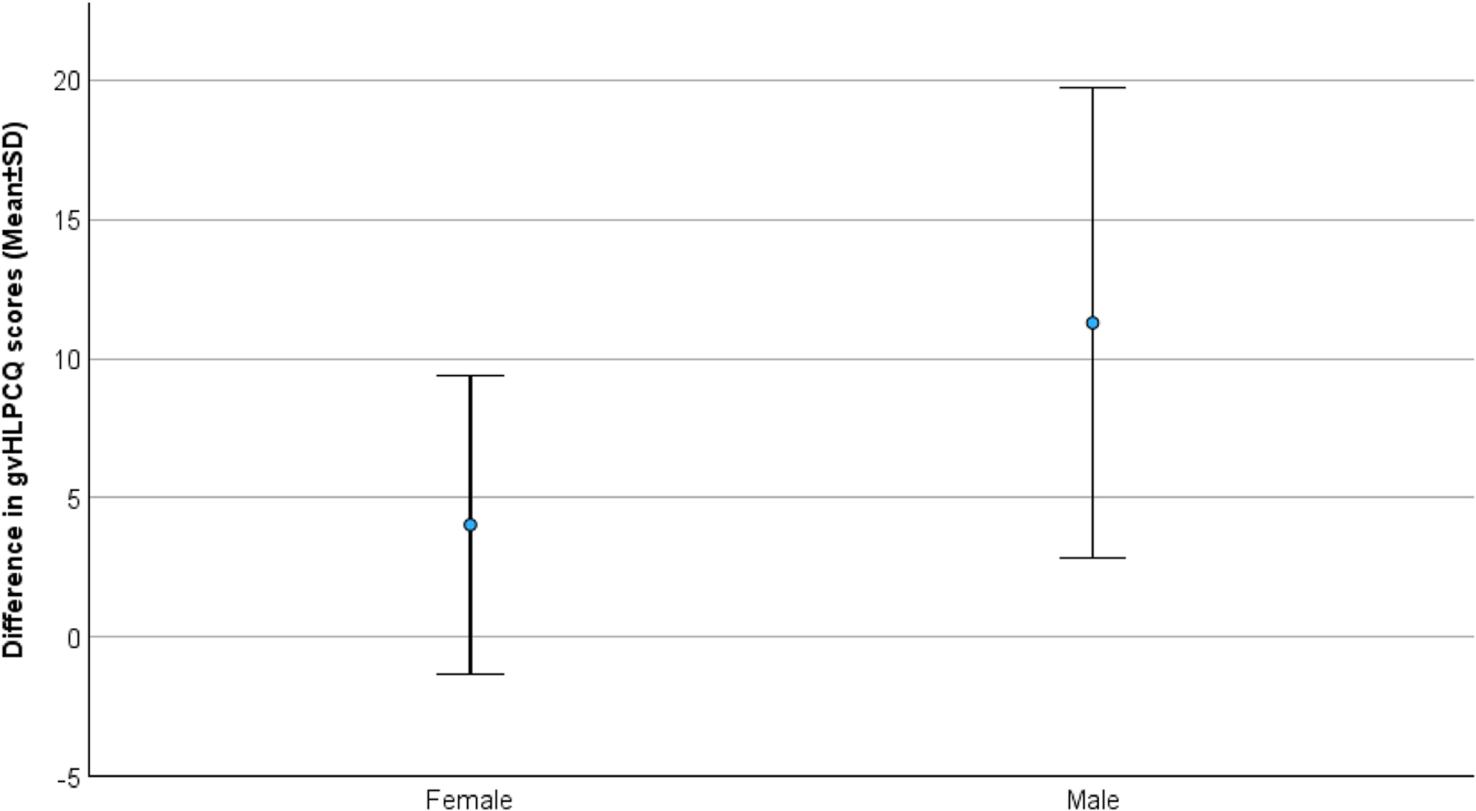
Improvement of the mean gvHLPCQ total Scores between 0 and 6 months. Abbreviations: gvHLPCQ German version of the Healthy Lifestyle and Personal Control Questionnaire, SD standard deviation

The results of the assessment of daily health habits and risk factors at 0 and 6 months are summarized in Table 2. The gvHLPCQ at 0 months demonstrated good reliability (Cronbach’s alpha 0.847). Change in the gvHLPCQ was the main endpoint of our study. The score was assessed at 0, 3, and 6 months. An increase in total gvHLPCQ was observed in the survey at 3 months and persisted until the survey at 6 months. Total gvHLPCQ scores increased significantly during the study period from 63.2 ± 11.1 at 0 months to 68.8 ± 10.7 at 6 months (mean change 5.6 points (95% CI 3.20 to 7.95); *p* < 0.001; Fig. 3a). We found significant increases in all subcategories of the gvHLPCQ in the same period, except for the subcategory of “Social and Mental Balance” (*p* = 0.290). We found a strong effect size, particularly for the total score (Cohen’s d 0.833) and the subcategories of “Dietary Health Choices” (Cohen’s d 0.827) and “Daily Routine” (Cohen’s d 0.579). We found that gvHLPCQ scores at 0 months (RCB − 0.222 [CI −0.427 to −0.018], *p* = 0.034), male gender (RCB 7.247 [CI 1.971 to 12.524], *p* = 0.009;), and scores in “Dietary Health Choices” at 0 months (RCB − 0.853 [CI −1.424 to −0.282], *p* = 0.005) were strongly associated with improvement in gvHLPCQ total scores at 6 months on linear univariate regression analysis. Male gender was most strongly associated with lower scores in the subcategory of “Dietary Health Choices” at 0 months (RCB − 4.643 [CI −7.409 to −1.877], *p* = 0.002). Male participants had an increase in the total gvHLPCQ Score of 11.3 ± 8.5 versus 4.0 ± 5.3 in female participants (*p* = 0.066; Fig. 4) and generally had lower gvHLPCQ scores at 0 months (57.9 ± 12.8 versus 64.3 ± 10.3; *p* = 0.165), however these results did not reach statistical significance. Male participants had significantly lower scores in the subcategory of “Dietary Health Choices” (12.4 ± 2.8 versus 17.1 ± 3.3, *p* = 0.002) and higher scores in the subcategory of “Social and mental balance” at 0 months (15.1 ± 2.6 versus 12.7 ± 2.9; *p* = 0.049).

**Table 2:** Comparison of the main outcome parameters at 0 and at 6 months

Item	Pairs	Mean ± SD examination at 0 months	Mean ± SD examination at 6 months	Mean difference (95% CI)	Paired t-test: *p*-value	Cohen’s d
gvHLPCQ total score	33	63.2 ± 11.1	68.8 ± 10.7	5.6 (3.20 to 7.95)	< 0.001	0.833
gvHLPCQ: Dietary Health Choices	33	16.1 ± 3.8	18.1 ± 3.3	2.0 (1.16 to 2.90)	< 0.001	0.827
gvHLPCQ: Dietary Harm Avoidance	33	11.0 ± 2.6	11.8 ± 2.4	0.8 (0.07 to 1.50)	0.031	0.392
gvHLPCQ: Daily Routine	33	18.9 ± 5.0	20.7 ± 4.8	1.8 (0.69 to 2.88)	0.002	0.579
gvHLPCQ: Organized Physical Exercise	33	4.0 ± 1.9	4.7 ± 1.6	0.6 (0.18 to 1.10)	0.008	0.492
gvHLPCQ: Social and Mental Balance	33	13.2 ± 2.9	13.5 ± 3.1	0.3 (−0.30 to 0.96)	0.290	0.187
Systolic blood Pressure (mmHg)	33	131.1 ± 14.0	135.6 ± 15.5	4.5 (0.22 to 8.75)	0.040	0.373
Weight (kg)	33	76.1 ± 15.4	74.4 ± 13.5	−1.7 (−3.22 to −0.10)	0.038	0.376
Abdominal circumference (cm)	33	96.2 ± 14.0	93.4 ± 11.1	−2.8 (−5.28 to −0.25)	0.032	0.390
BMI (kg/m²)	33	26.2 ± 4.9	25.6 ± 3.9	−0.6 (−1.17 to −0.02)	0.044	0.365
LDL-cholesterol (mg/dl)	34	120.9 ± 32.4	116.2 ± 35.6	−4.7 (−14.25 to 4.90)	0.327	0.170
HbA1c (%)	34	5.4 ± 0.6	5.4 ± 0.5	0.0 ± 0.2	0.848	0.033
5-year estimated stroke risk (%)	32	2.5 ± 1.9	2.2 ± 1.8	−0.3 (−0.52 to −0.04)	0.025	0.415
10-year estimated stroke risk (%)	32	5.1 ± 4.3	4.8 ± 4.3	−0.2 (−0.07 to 0.18)	0.250	0.207

The Stroke Riskometer™ Score was used to estimate the effects of our intervention on the 5- and 10-year estimated stroke risk. We found significant decreases in the 5-year estimated stroke risk from 2.5% to 2.2% (mean difference − 0.3 (95% CI −0.52 to −0.04); *p* = 0.025). Similar absolute decreases were seen for the 10-year estimated stroke risk from 5.1% to 4.8%, although the reduction was not statistically significant (*p* = 0.250) (see Table 2).

#### Impact on objectifiable risk factors (lab values, abdominal circumference and BMI) and additional results

Objectifiable risk factors were assessed in a standardized medical examination at 0 and at 6 months (for details see Table 2): We did not detect significant decreases in LDL cholesterol (*p* = 0.327; Fig. 3b) or the HbA1c (*p* = 0.848), despite a notable trend towards decreasing cholesterol levels. Importantly, weight (mean difference − 1.7 kg (95% CI −3.22 to −0.10); *p* = 0.038), abdominal circumference (mean difference − 2.8 cm (95% CI −5.28 to −0.25); *p* = 0.032; Fig. 3c), and BMI (mean difference − 0.6 kg/m² (95% CI −1.17 to −0.02); *p* = 0.044; Fig. 3d) all decreased significantly during the study period. Systolic blood pressure increased slightly during the study period (*p* = 0.040).

Additional secondary endpoints were assessed through individual survey items: We found that participants significantly increased their weekly intake of fish (*p* = 0.001). Changes in daily vegetable (*p* = 0.317) and alcohol consumption (*p* = 0.257) were not statistically significant. Participants also indicated that they tended to increase the overall number of hours spent on physical exercise per week (*p* = 0.057) in addition to significant increases (*p* = 0.008) in the subcategory of organized physical exercise of the gvHLPCQ. Of the three participants with regular tobacco consumption at the beginning of the study, 2 had quit smoking altogether at 6 months. Participants also reported travelling car-free more often after 6 months (*p* = 0.032).

#### User profiles and participant feedback

**Fig. 5:**
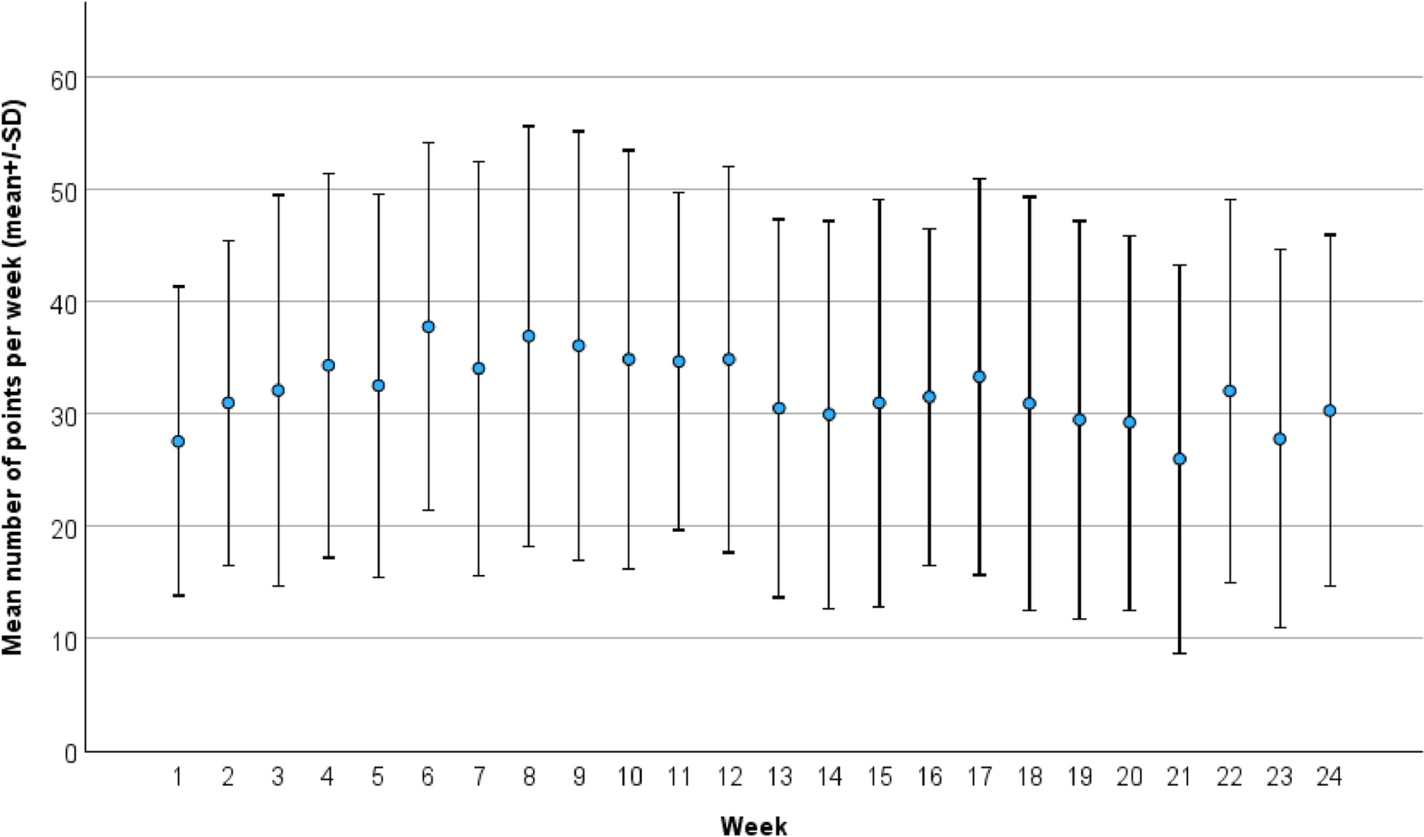
Mean number of points gathered by individual participants per week until the final physical examination

Participants earned a total of 25,355 points and a mean of 975.2 points per week during the study period. We observed that participation remained consistently high throughout the study, with a slight decrease in the number of points gathered in the last months of the study (Fig. 5). Most points were gathered through scheduled activities (36.1%), followed by the 6,000 (15.1%) and 8,000 (11.1%) step goals. The largest shares of the 9,196 points gathered in the category of scheduled activities were “taking walks” (37.6%), “self-defined activities” (22.1%), “cycling” (19.0%), and “weight training” (11.0%).

Overall, participant feedback was positive, with 14 participants (42.4%) indicating that the study helped them “much” or “very much” to improve their health habits. Only 1 participant (2.8%) did not believe that participation provided any benefit. At six months, male participants reported a greater increase in healthy behaviors from participating in this study compared to female participants (*p* = 0.012). When asked about which aspects of the study were most beneficial, respondents indicated that the MAPS app (median 3 [IQR 2–3]; “much”) provided the greatest benefit. Information given at the beginning of the study and the individual examinations were considered equally beneficial (median 2 [IQR 2–3], “somewhat”). Feedback provided after each examination concerning individual risk profiles was considered to have been least helpful (median 2 [IQR 1.25–3.25], “somewhat”). Concerning individual functions of the MAPS app, participants indicated that the pedometer function (median 3 [IQR 2–4]; “much”), as well as the reward system (median 3 [IQR 2–3], “much”), had been the most popular, followed by the comparison to other participants, podcasts, weekly weight function, and nutrition goal (median 2 [IQR 2–3]; “somewhat”). The reminder function of the app was considered the least helpful (median 2 [IQR 1–3], “somewhat”).

## Discussion

### Main findings

1. We observed statistically significant improvements in the gvHLPCQ scores, significant reductions in the 5-year estimated stroke risk, as well as reductions in the calculated BMI and abdominal circumference at 6 months.
2. Changes in LDL cholesterol and HbA1c levels did not reach statistical significance. Similarly, several additional items, such as alcohol consumption and the number of hours spent on physical exercise per week, showed signs of improvement without reaching statistical significance.
3. Male participants constituted only 20% but tended to have lower initial gvHLPCQ scores while experiencing greater improvement during the study period in their daily health habits. Future research should concentrate on understanding how health-promoting interventions affect different genders in distinct ways.

### Discussion of the main findings and comparison to previous studies

1) We observed improvements in gvHLPCQ scores at 3 months that were maintained until the end of the trial at 6 months. The average points scored on the app per week remained mostly unchanged throughout the study period, and we found no evidence of habituation. Along with improvements in mean BMI and abdominal circumference, this indicates that the MAPS app was effective in enhancing daily habits and reducing objective risk factors for cerebrovascular diseases.

We demonstrated significant improvements in the 5-year stroke risk using the SRA™. However, improvements in the 10-year stroke risk were not statistically significant. This discrepancy can be explained by the increased proportion of the non-modifiable stroke risk factors, particularly age, that have more weight in assessments of long-term stroke risk. While statistically significant, this improvement may be clinically meaningful only if the MAPS app is widely adopted, as the absolute risk reduction was limited. However, we anticipate that the impact of our intervention may be more pronounced in the general population, as most participants in this study were healthier and more health-conscious than the average individual. Studies have examined whether the SRA™ can reduce stroke risk, as the application analyzes individual risk profiles and provides relevant suggestions. These features were not used in our study. So far, the use of the SRA™ has not been shown to conclusively reduce stroke risk by itself, despite being an effective tool to improve awareness of stroke and its risk factors [23, 24]. We found one intervention study that demonstrated a reduction in estimated stroke risk using the SRA™ [25]. The absolute risk reduction in that study was by 0.23%, which is similar to the reduction observed in our study. The results of the phase III interventional PERKS-Trial on the efficacy of the SRA™ to reduce stroke risk have yet to be published [26]. 

2) LDL cholesterol, HbA1c and systolic blood pressure did not decrease significantly during this study, although we observed a trend toward lower LDL cholesterol values after 6 months. We believe that this is mostly due to normal or near-normal mean values for all three risk factors at enrollment. This suggests that participants were more health-conscious than the general population. This was also reflected by the relatively low proportion of smokers in our sample (8.6% compared with 20.1% in the adult population) and the lower-than-average mean BMI values (26.2 compared to an estimated 26.4 for 50–55-year-olds in the general population) [27, 28]. We observed a statistically significant increase in systolic blood pressure after six months. However, we believe this increase is due to normal fluctuations in blood pressure rather than an effect of our intervention. Moreover, we observed very consistent improvements in several health habits. Even when differences were non-significant, we observed robust trends towards healthier behaviors in our participants (i.e. more physical exercise, eating more portions of vegetables, etc.). These improvements may reach statistical significance with a larger sample size, which should be confirmed by a larger randomized controlled follow-up study.

3) Male participants constituted only 20% of our sample. Previous studies reported that women are more interested in and seek health-related information more actively than men [29]. Although women are typically underrepresented in randomized controlled trials, their participation rate is typically higher in primary prevention studies. A study on primary stroke prevention using the SRA™ also reported that 60% of participants were women [24]. Despite the relatively small proportion of male participants in our study, we found statistically significant differences between male and female participants. The greatest association of gender was seen for lower scores in the subcategory “Dietary Health Habits” of the gvHLPCQ for males. This aligns with earlier studies on gender-specific dietary habits and physical activity [30]. Men have been found to have unhealthier dietary habits and to consume more alcoholic beverages, while women are more likely to avoid unhealthy foods due to perceived unhealthiness [31, 32]. Male participants also tended to have lower gvHLPCQ scores at enrollment and experienced greater improvement in this score during this study, although these differences did not reach statistical significance. Our findings suggest that although males may be less interested in participating in primary prevention studies, they may gain more from participation in such programs, particularly in interventions targeting dietary habits. Future studies should encourage greater male participation in larger primary prevention studies to confirm our observations.

### Lessons learned and recommendations for future health apps

Our MAPS app features a user-friendly design that integrates well-known principles of behavioral therapy to help users develop healthy habits. The app is designed to simultaneously improve various cerebrovascular risk factors by utilizing reminder functions, user feedback, and gamification elements. This unique combination distinguishes the app. Based on the feedback we received from study participants, the use of a mobile app was found to be an effective tool for encouraging healthy behavior. Participants generally responded more to positive feedback and encouragement and reward functions and were less likely to respond to reminder functions. The pedometer function of the MAPS app was estimated to have been the most helpful. We found the six-month intervention period to be sufficient to assess persistent changes in health habits. Based on feedback from our participants, future health-promoting apps should be developed around an easily accessible and visible pedometer function with positively formulated and motivating push messaging and reward concepts.

### Strengths and limitations

The main limitation of our study is the small sample size and the lack of a control group, which restricts our ability to conclusively demonstrate the effectiveness of our intervention. The multicomponent design indicates that outcomes may have been affected by factors such as feedback after examinations and participant training, in addition to the MAPS app. Participants were recruited from a single workplace, and participation was limited to Android users, which limits the generalizability of our findings. However, this was intended as a pilot study, primarily designed to identify significant changes in the gvHLPCQ score and to demonstrate the feasibility of our study design. We were not adequately powered to assess significant changes in other measurable risk factors, such as LDL cholesterol and HbA1c; larger sample sizes would be required to detect clinically relevant changes in these areas. We did not perform validation or reliability testing of the translated version of the HLPCQ and the SRA™ questions, which may limit the reliability of our results. Data collection depended on whether participants carried their smartphones or chose to use additional wearable devices, which resulted in relevant inter-individual differences in step counts. Additionally, the sample exhibited inherent selection bias due to voluntary participation in the study. Most participants were health-conscious and had overall lower risk profiles. These findings must also be interpreted considering the self-reported nature of the data, which introduces the possibility of a social desirability bias. Future randomized trials should investigate the effectiveness of the MAPS app in larger populations with a control group for comparison.

## Conclusion

This exploratory pilot study presents initial evidence that the MAPS app may promote healthier daily habits with the potential to reduce the risk of stroke. While these findings are promising, they remain preliminary and require further validation in larger, rigorously designed randomized controlled trials. Additionally, our results suggest potential gender-specific responses, which warrant further investigation.

## Supplementary Information

Supplementary Material 1.

## Data Availability

Data will be made available by the corresponding author upon reasonable request.

## Abbreviations

BMI: Body Mass Index
CI: Confidence interval
gvHLPCQ: German version of the HLPCQ
HLPCQ: Healthy Lifestyle and Personal Control Questionnaire
HbA1c: Hemoglobin A1c
IQR: Interquartile range
LDL: Low-density lipoprotein
MAPS: Mobile Health Application for the Prevention of Stroke
RCB: Regression Coefficient B
SRA™: Stroke Riskometer App™
