# Supplementary material for: Mobile Health Application for the Prevention of Stroke (MAPS): a pilot, single-arm study on an innovative health application designed for primary stroke prevention
Source: BMC Neurol. 2026 Jan 10;26:56. doi: 10.1186/s12883-025-04560-3 (PMC12853848; doi:10.1186/s12883-025-04560-3)
Supplement: Supplementary file 1 — Supplementary Material 1. [file 12883_2025_4560_MOESM1_ESM.pdf]

Supplemental table S1: Questionnaire at baseline (Questions 7 to 32 are translations of the gvHLPCQ back into English, making them nearly identical to the original HLPCQ published by Darviri et al.(1))

Participant number:

Please only select one of the possible answers. Completion time: Approx. 10-15 minutes.

Question 1	Full-time	Part-time	Retired	None
What is your current employment status?				

Question 2	None	High School	Vocational training	University degree
What is your highest level of education?				

Question 3	None	<1	1-2	>2
How many alcoholic beverages do you consume daily?				

Question 4	None	≤1	1-2	≥2.5
How many hours do you exercise per week				

Question 5	None	≤1	2-3	4-5	≥6
How many portions of vegetables/fruit do you consume daily?					

Question 6	None	1-5	6-10	11-15	16-20	>20

How many cigarettes do you smoke daily?						
	Never		Stopped >12 months ago		Stopped <12months ago	
Have you ever smoked?						

Question 7	Never/Rarely	Sometimes	Often	Always
Do you pay attention to how much food you put on your plate?				

Question 8	Never/Rarely	Sometimes	Often	Always
Do you check food labels before buying food?				

Question 9	Never/Rarely	Sometimes	Often	Always
Do you calculate the calories in your meals?				

Question 10	Never/Rarely	Sometimes	Often	Always
Do you limit fat in your meals?				

Question 11	Never/Rarely	Sometimes	Often	Always
Do you cook your own food?				

Question 12	Never/Rarely	Sometimes	Often	Always
Do you prefer organic food?				

Question 13	Never/Rarely	Sometimes	Often	Always
Do you prefer whole grain products?				

Question 14	Never/Rarely	Sometimes	Often	Always
Do you avoid packaged goods or fast food?				

Question 15	Never/Rarely	Sometimes	Often	Always
Do you avoid sodas or sugary drinks (soft drinks)?				

Question 16	Never/Rarely	Sometimes	Often	Always
Do you avoid eating when you are stressed or disappointed?				

Question 17	Never/Rarely	Sometimes	Often	Always
Do you avoid binge eating when you're out with friends?				

Question 18	Never/Rarely	Sometimes	Often	Always
Do you eat your meals at the same time every day?				

Question 19	Never/Rarely	Sometimes	Often	Always
Do you make sure you don't miss any meals?				

Question 20	Never/Rarely	Sometimes	Often	Always
Do you have a healthy breakfast?				

Question 21	Never/Rarely	Sometimes	Often	Always
Do you go to bed at the same time every day?				

Question 22	Never/Rarely	Sometimes	Often	Always

Do you follow a set schedule for your daily activities?				

Question 23	Never/Rarely	Sometimes	Often	Always
Do you eat breakfast at the same time every day?				

Question 24	Never/Rarely	Sometimes	Often	Always
Do you eat lunch at the same time every day?				

Question 25	Never/Rarely	Sometimes	Often	Always
Do you eat dinner at the same time every day?				

Question 26	Never/Rarely	Sometimes	Often	Always
Do you do endurance sports for 20 or more minutes at least 3 times a week?				

Question 27	Never/Rarely	Sometimes	Often	Always
Do you follow a regular training plan to exercise?				

Question 28	Never/Rarely	Sometimes	Often	Always
Do you share your personal problems or worries with others?				

Question 29	Never/Rarely	Sometimes	Often	Always
Do you focus on positive thoughts during difficult times?				

Question 30	Never/Rarely	Sometimes	Often	Always
Can you fall asleep without worries or thoughts about the next day?				

Question 31	Never/Rarely	Sometimes	Often	Always
Is it important to you to meet with your family every day and exchange ideas with them?				

Question 32	Never/Rarely	Sometimes	Often	Always
Do you organize your time in such a way that work, private life, and leisure are in balance?				

Question 33	Never/Rarely	Sometimes	Often	Always
Are you walking or cycling to a destination that you could				

otherwise more easily reach by car or public transport?				

Question 34	Never	<1	1-2	>2
How often a week do you eat fish?				

Question 35	Worse	Similar	Better	Much better
How would you rate your lifestyle compared to your peers of the same age and socioeconomic status?				

Question 36	Yes	No
Did you experience any extraordinary stress or depressive episodes in the past year?		

Question 37	Yes	No
Did a parent suffer a stroke or heart attack before reaching the age of 65?		

Question 38	Yes	No
Are you taking any medication to lower your blood pressure?		

Question 39	Yes	No
Are you taking any cholesterol-lowering or lipid-lowering medications?		

Question 40	No	Yes, more than 12 months ago	Yes, within the last 12 months
Have you been diagnosed with diabetes?			

Question 41	No	Yes, more than 12 months ago	Yes, within the last 12 months
Have you been diagnosed by a doctor with a heart condition (chest pain, heart attack, heart failure) or peripheral arterial disease (PAD)?			

Question 42	No	Yes, more than 12 months ago	Yes, within the last 12 months
Have you been diagnosed with an enlarged heart (heart failure/cardiomyopathy) by a doctor?			

Question 43	No	Yes, more than 12 months ago	Yes, within the last 12 months
Have you been diagnosed with atrial fibrillation by a doctor?			

Question 44	No	Yes, more than 12 months ago	Yes, within the last 12 months
Have you been medically diagnosed with dementia or cognitive impairment?			

Question 45	Yes	Never
Do you or someone close to you believe that you have a bad memory?		

Question 46	No	Yes, more than 12 months ago	Yes, within the last 12 months
Have you been diagnosed with traumatic brain injury by a doctor?			

Question 47	No	Yes, more than 12 months ago	Yes, within the last 12 months
Have you been diagnosed by a doctor with a stroke or a transient ischemic attack (TIA)?			

Question 48	Yes	No
Have you already used other health-promoting apps or “fitness apps”?		

Question 49	<2	2-4	5-7	>7
How many hours a day do you carry your mobile phone on your person (either directly on your body or in your backpack/handbag)?				

Question 50	Never/Rarely	Sometimes	Often	Very often
To what extent do the following factors prevent you from behaving in a healthier way?				
Lack of motivation				
Lack of time				
Uncertainties about how to implement healthy behavior				
Procrastination (postponing)				
Negative habits outweigh positive ones (e.g., snacks)				

Question 51	Yes	No
Do you use an external pedometer (e.g., a fitness watch)?		

1. Darviri C, Alexopoulos EC, Artemiadis AK, Tigani X, Kraniotou C, Darvyri P, et al. The Healthy Lifestyle and Personal Control Questionnaire (HLPCQ): a novel tool for assessing self-empowerment through a constellation of daily activities. BMC Public Health. 2014 Dec;14(1):1–10.
